# MicroRNA Involvement in Signaling Pathways During Viral Infection

**DOI:** 10.3389/fcell.2020.00143

**Published:** 2020-03-10

**Authors:** Madalina Gabriela Barbu, Carmen Elena Condrat, Dana Claudia Thompson, Oana Larisa Bugnar, Dragos Cretoiu, Oana Daniela Toader, Nicolae Suciu, Silviu Cristian Voinea

**Affiliations:** ^1^Alessandrescu-Rusescu National Institute for Mother and Child Health, Fetal Medicine Excellence Research Center, Bucharest, Romania; ^2^Department of Cell and Molecular Biology and Histology, Carol Davila University of Medicine and Pharmacy, Bucharest, Romania; ^3^Division of Obstetrics, Gynecology and Neonatology, Carol Davila University of Medicine and Pharmacy, Bucharest, Romania; ^4^Department of Obstetrics and Gynecology, Alessandrescu-Rusescu National Institute for Mother and Child Health, Polizu Clinical Hospital, Bucharest, Romania; ^5^Department of Surgical Oncology, Institute of Oncology Prof. Dr. Alexandru Trestioreanu, Carol Davila University of Medicine and Pharmacy, Bucharest, Romania

**Keywords:** microRNA, signaling, viral, HPV, HIV, hepatitis, herpes

## Abstract

The study of miRNAs started in 1993, when Lee et al. observed their involvement in the downregulation of a crucial protein known as LIN-14 in the nematode *Caenorhabditis elegans.* Since then, great progress has been made regarding research on microRNAs, which are now known to be involved in the regulation of various physiological and pathological processes in both animals and humans. One such example is represented by their interaction with various signaling pathways during viral infections. It has been observed that these pathogens can induce the up-/downregulation of various host miRNAs in order to elude the host’s immune system. In contrast, some miRNAs studied could have an antiviral effect, enabling the defense mechanisms to fight the infection or, at the very least, they could induce the pathogen to enter a latent state. At the same time, some viruses encode their own miRNAs, which could further modulate the host’s signaling pathways, thus favoring the survival and replication of the virus. The goal of this extensive literature review was to present how miRNAs are involved in the regulation of various signaling pathways in some of the most important and well-studied human viral infections. Further on, knowing which miRNAs are involved in various viral infections and what role they play could aid in the development of antiviral therapeutic agents for certain diseases that do not have a definitive cure in the present. The clinical applications of miRNAs are extremely important, as miRNAs targeted inhibition may have substantial therapeutic impact. Inhibition of miRNAs can be achieved through many different methods, but chemically modified antisense oligonucleotides have shown the most prominent effects. Though scientists are far from completely understanding all the molecular mechanisms behind the complex cross-talks between miRNA pathways and viral infections, the general knowledge is increasing on the different roles played by miRNAs during viral infections.

## Introduction

MicroRNAs are small molecules of non-coding RNAs, which contain from 17 to 25 nucleotides (Wang et al., 2016) and exert their functions by modulating gene expression (Macfarlane and Murphy, 2010; Wahid et al., 2010). They were first described in 1993 by Lee et al., who observed that microRNAs downregulated a crucial protein known as LIN-14, involved in the progression of the nematode *Caenorhabditis elegans* from L1 to L2 larval stage (Lee et al., 1993; Bhaskaran and Mohan, 2014). Since then, great progress has been made regarding research on microRNAs, which are now known to be involved in the regulation of various physiological and pathological processes in both animals and humans.

The biogenesis of microRNAs is a dynamic process, involving a multitude of mechanisms that will finally result in the formation of mature miRNAs (Ketting, 2010). Any disrupting event that appears on this pathway could lead to an increased or decreased production of miRNAs in the targeted tissue, leading to various diseases such as neoplasia, ischemic heart disease, hematological diseases, muscular dystrophies, neurodegenerative diseases, psychiatric disorders, brain tumors, kidney disease, etc., according to the physiological functions regulated by the impaired miRNA (Sayed and Abdellatif, 2011; Garofalo et al., 2014; Trionfini et al., 2015; Barwari et al., 2016; Luoni and Riva, 2016).

The process of miRNA formation begins in the nucleus, with the transcription of the miRNA genes, by RNA polymerase II (Pol II), resulting in a “hairpin” structured primary transcript encoding miRNA sequences (Ha and Kim, 2014). This step is positively or negatively regulated by RNA Pol II-associated transcription factors like p53, ZEB1 and ZEB2, MYC and also by epigenetic modulators such as the methylation of DNA and histone modification (Lee et al., 2004; Davis-Dusenbery and Hata, 2010; Krol et al., 2010; Ha and Kim, 2014). Further on, the primary miRNA (pri-miRNA) goes through a series of maturation processes, the first one taking place in the nucleus. At this point, RNase III Drosha along with the co-factor DGCR8 forms the Microprocessor complex, which crops the loop end of pri-miRNA, forming precursor miRNA which also has a “hairpin”-like structure (pre-miRNA) (Denli et al., 2004; Gregory et al., 2004; Han et al., 2004).

The resulting product is exported by Exportin-5 into the cytoplasm to undergo the following steps for maturation (Ha and Kim, 2014). There, the pre-miRNA is once again cropped near the loop end by another RNase named Dicer, resulting in a small RNA duplex (Ketting et al., 2001; Knight and Bass, 2001; Hutvagner et al., 2001). Further on, the generated product forms, with an argonaute (AGO) protein, the RNA-induced silencing complex (RISC) (Hammond et al., 2001).

The most important roles that miRNAs have are gene regulation and intercellular signaling (O’Brien et al., 2018). For the first one, the miRISC can work through two mechanisms known as canonical, or most frequently used, the non-canonical mechanism (Bartel, 2009; Helwak et al., 2013; Chevillet et al., 2014; Eichhorn et al., 2014; Kai et al., 2018). The canonical mode of action involves the binding of miRISC to the 3′-untranslated region (3′-UTR) of the targeted mRNA, leading to a cessation of translation when the two strains are almost completely complementary, or to a decrease in translation when the complementarity is limited (Reinhart et al., 2000; Dalmay, 2008; Sand, 2014). The non-canonical pathway does not require such high complementarity (Helwak et al., 2013). Early studies determined that within the seed region, only 6-nt matches were required in order to obtain a functional miRNA - targeted mRNA interaction (Bartel, 2009). As a result, the canonical sites were defined as it follows: 3 possible canonical sites for 6-mers matches to positions 1-6, 2-7, and 3-8, 2 possible canonical sites for 7- mers matches to positions 2-8 and 1-7, and one possible canonical site for 8-mer match, to position 1-8 (Lee et al., 1993; Wightman et al., 1993; Poy et al., 2004).

In contrast, it has been found that miRISC complexes, targeting sites different from the seed region, with a diminished complementarity, do exert some modest regulatory functions (Helwak et al., 2013). Examples of non-canonical sites are “nucleation bulges,” formed from 5 consecutive nucleotides, located on positions 2-6, which following the “pivot pairing rule” proposed by Chi et al. in 2012, pair themselves in position 6 of the miRNA, known as “pivot” (Chi et al., 2012; Seok et al., 2016). It is apparent that many miRNAs studied have a pivot nucleotide able to bind a nucleation bulge. For example, miR-124 has a C nucleotide in position 6 that would bind a complementary G-bulge, let-7 has a U pivot nucleotide and miR-708 contains a G pivot nucleotide (Chi et al., 2012). “Seed-like motifs” are also non-canonical binding sites found on targeted mRNA (Seok et al., 2016). These sites seem to have certain mismatches or deletions that demand additional 3′ interactions in order to make the association functional (Seok et al., 2016). However, the non-canonical pathways have not been thoroughly researched and their exact functions are still to be determined.

MiRNAs have also been shown to have a role in viral infections. As we already know, viruses are intracellular organisms that solely rely on the host environment to multiply (Girardi et al., 2018). It has been observed that these pathogens can induce the up-/downregulation of various host miRNAs in order to elude the host’s immune system (Scheel et al., 2016; Girardi et al., 2018; Shimakami et al., 2012; Trobaugh et al., 2014). In contrast, some miRNAs studied could have an antiviral effect, enabling the defense mechanisms to fight the infection or, at the very least, they could induce the pathogen to enter a latent state (Guo et al., 2013; O’Connor et al., 2014; Pan et al., 2014; Girardi et al., 2018). Knowing which miRNAs are involved in various viral infections and what role they play could aid in the development of antiviral therapeutic agents for certain diseases that do not have a definitive cure in the present.

## Classification of MicroRNAs Involved in Viral Infections

Although we only presented host miRNAs so far, we have to acknowledge that some viruses can also synthesize their own miRNAs (Pfeffer et al., 2004). As a result, in the beginning of our classification we could divide them into host miRNAs and viral miRNAs, according to their source (Table 1). Similar to host miRNAs, the viral ones participate in the life cycle of the virus and could induce certain modifications in the host cells (Guo et al., 2015; Bruscella et al., 2017). Likewise, they are also processed by the previously mentioned RNases Drosha and Dicer, although some viral miRNAs have been described to skip the first step and pass directly to Dicer processing (Bogerd et al., 2010; Diebel et al., 2010; Tycowski et al., 2015).

**TABLE 1:** Classification of microRNAs based on their source and roles.

Classification criteria	
Source	Host microRNAs
	Viral microRNAs
Roles	*Host microRNAs:*
	Proviral microRNAs
	Antiviral microRNAs

Regarding the role they play, host miRNAs could be classified as proviral or antiviral according to their actions once a certain virus has entered the host cell. Through the interaction between viral and host miRNAs, the latter could enable the viral replication and infection, thus exerting a proviral function (Masaki et al., 2015; Scheel et al., 2016; Bruscella et al., 2017). Furthermore, proviral miRNAs can promote viral infection by suppressing antiviral factors, such as interferon (IFN), allowing the virus to escape the immune response of the host (Sharma et al., 2015; Bruscella et al., 2017). In contrast, various host miRNAs can act have antiviral functions by influencing the production of viral RNA, blocking the viral replication, suppressing proviral proteins or by inducing the virus to enter a latent phase (Zheng et al., 2013; Wu et al., 2014; Slonchak et al., 2015; Ho et al., 2016).

A more general classification, valid for all miRNAs, not only for those involved in viral infection, would be based on the state in which they can be found in body fluids. Their presence has been detected in all biological fluids, among them being blood, tears, urine, amniotic fluid, breast milk, semen and saliva (Turchinovich et al., 2012). Thus, approximately 90% of the extracellular or circulating miRNAs can be found associated with AGO proteins and the other 10% is transported in microvesicles like exosomes and apoptotic bodies (Turchinovich et al., 2013). Both proteins and microvesicles protect the miRNAs they carry from the degradation of RNases and confer them a high stability in an unfavorable extracellular environment (Turchinovich et al., 2013). A comprehensive list of the functions and pathways targeted by each miRNA described in this manuscript can be found in Table 2.

**TABLE 2:** Classification, functions and targeted signaling pathways by miRNA.

miRNA	Source	Characteristics	Pathway(s) targeted	References
miR-34 family	Host	– Repressor of Wnt pathway	Wnt signaling pathway	Kim et al., 2011; Cha et al., 2012; Smith et al., 2017
		– Antiviral functions – especially on flaviviruses		
miR-155	Host	– Stimulator of Type I Interferon signaling pathway	Type I Interferon signaling pathway	Jiang et al., 2010; Wang P. et al., 2010; Yao et al., 2012; Zhang et al., 2012; Li et al., 2013; Ruelas et al., 2015
		– Increases the innate and adaptive immune response		
			NF-κB signaling pathway Wnt/β-catenin signaling	
		– Antiviral functions		
		– Inhibitor of the HBV replication		
		– Higher levels in HIV infected cells		
		– Inhibition of NF-κB signaling pathway – promotion of viral latency		
		– Higher levels in HCV infection → increase the activity of		
miR-19a	Host	– Stimulator of Type I Interferon signaling pathway	Type I Interferon signaling pathway	Pichiorri et al., 2008; Jiang et al., 2010; Wang P. et al., 2010; Su et al., 2011; Yao et al., 2012; Li et al., 2013
		– Increases the innate and adaptive immune response		
		– Antiviral functions		
miR-122	Host	– Stimulator of Type I Interferon signaling pathway	Type I Interferon signaling pathway	Pichiorri et al., 2008; Kalluri and Weinberg, 2009; Jiang et al., 2010; Wang P. et al., 2010; Chen Y. et al., 2011; Su et al., 2011; Jopling, 2012; Wang et al., 2012; Xu et al., 2012; Yao et al., 2012; Li et al., 2013
		– Increases the innate and adaptive immune response		
		– Antiviral functions	Wnt/β-catenin pathway	
			RhoA/Rock pathway	
		– Liver-specific		
		– Antiviral functions in HBV infection – Inhibition of HBV replication		
		– Diminished levels — promotion of viral persistence and oncogenesis		
		– Antitumorigenic effects by regulation of Wnt/β-catenin pathway		
		– Impairment of RhoA/Rock pathway		
let-7 family	Host	– Decreased expression in Kaposi sarcoma associated to herpesvirus and HIV infections	NF-κB signaling pathway	Androulidaki et al., 2009; Shishodia et al., 2014
			STAT3 signaling pathway	
		– Their low levels increase the expression of the NF-κB signaling pathway → increased inflammation		
		– Increasing the level of let-7a → decreased STAT3 supplies		
miR-233	Host	– Decreased expression in Kaposi sarcoma associated to herpesvirus and HIV infections	NF-κB signaling pathway	Androulidaki et al., 2009
		– Their low levels increase the expression of the NF-κB signaling pathway → increased inflammation		
miR-146	Host	– Increased in HIV and HCV infections	NF-κB signaling pathway	Bhaumik et al., 2008; Houzet et al., 2008
		– Decreased activity of the NF-κB signaling pathway		
		– Proviral functions		
miR-21	Host	– Increased in HIV and HCV infections	NF-κB signaling pathway	Bhaumik et al., 2008; Houzet et al., 2008; Shishodia et al., 2014; He et al., 2019
		– Decreased activity of the NF-κB signaling pathway		
			MAP2K3/p38 MAPK pathway	
		– Proviral functions in HIV and HCV infection		
		– Antiviral functions in Coxsackievirus B3 infection	STAT3 signaling pathway	
		– Decreasing its level in HPV infections → downregulation of STAT3		
miR-218-5p	Host	– Down-regulation of NF-κB signaling pathway in HPV induced cervical cancer	NF-κB signaling pathway	Xu et al., 2018
hsa-miR-483-3p	Host	– Up-regulated by HCV	PI3K/Akt signaling pathway	Shwetha et al., 2013
		– Increase the activity of PI3K/Akt signaling pathway, prolonging cell survival		
		– Proviral functions		
hsa-miR-320c	Host	– Up-regulated by HCV	PI3K/Akt signaling pathway	Shwetha et al., 2013
		– Increase the activity of PI3K/Akt signaling pathway, prolonging cell survival		
		– Proviral functions		
miR-199a-5p	Host	– Proviral functions	PI3K/Akt signaling pathway	Wang et al., 2015
		– Its downregulation blocks the PI3K/Akt signaling pathway in HCV infection		
		– Low levels lead to a decrease of viral replication in HCV infection		
miR-125b	Host	– Suppressor of PI3K/Akt signaling pathway	PI3K/Akt signaling pathway	Cui et al., 2012; Wang et al., 2013; Banzhaf-Strathmann and Edbauer, 2014; Ribeiro and Sousa, 2014
		– Downregulated in HPV infection → limited cancer cell growth and increased apoptosis		
		– Higher levels → apoptosis inhibition		
miR-H4b	Viral — produced by HSV	– inhibition of PI3K/Akt signaling pathway and mTOR signaling pathways → better adaptation for viral replication and latency	PI3K/Akt signaling pathway mTOR signaling pathways	Gottwein and Cullen, 2008; Zhao et al., 2015
miR-744	Host	– Antiviral functions against RSV and influenza viruses	MAPK signaling pathway	McCaskill et al., 2017
miR-24	Host	– Antiviral functions against RSV and influenza viruses	MAPK signaling pathway	McCaskill et al., 2017
miR-124	Host	– Antiviral functions against RSV and influenza viruses	MAPK signaling pathway	McCaskill et al., 2017
miR-499a	Host	– Proviral functions in HCV infection	Notch signaling pathway	Sarma et al., 2012
miR-BART7-3p	Viral — produced by EBV	–Regulatory functions for the PI3K/Akt/GSK-3β pathway	PI3K/Akt/GSK-3β pathway	Cai L.M. et al., 2015; Wang et al., 2017
		– Aberant regulation of Wnt pathway → excessive cellular proliferation	Wnt signaling pathway	
miR-BART1	Viral — produced by EBV	– Activation of PI3K/Akt/GSK-3β pathway	PI3K/Akt/GSK-3β pathway	Cai L.M. et al., 2015
miR-BART16	Viral — produced by EBV	– Inhibition of IFN signaling pathway	IFN signaling pathway	Hooykaas et al., 2017
		– Proviral function → increased replication		
miR-BART19-3p, miR-BART17-5p, miR-BART14, miR-BART18-5p	Viral — produced by EBV	– Inhibition of Wnt pathway inhibitory genes	Wnt signaling pathway	Wong et al., 2012
miR-718	Host	– Upregulated in patients with both HIV and Kaposi sarcoma	PTEN/AKT/mTOR pathway	Xue et al., 2014
		– Inhibition of PTEN/AKT/mTOR pathway → inhibition of the tumor suppressor action of PTEN		
miR-942 miR-711	Host	– Upregulated in patients with both HIV and Kaposi sarcoma	NF-κB signaling pathway	Yan et al., 2018
		– Activation of NF-κB signaling pathway inhibition of the KSHV lytic replication		
miR-146a/b	Host	– Increased during viral infections	NF-κB signaling pathway	Taganov et al., 2006; Huang et al., 2018
		– Downregulation of NF-κB signaling pathway		
		– Proviral activity — increased inflammatory state, HIV persistence		
hiv1-miR-88 hiv1-miR-99	Viral — produced by HIV	– Activation of TLR8 signaling pathway	TLR8 signaling pathway	Silvestri and Feinberg, 2003; Bernard et al., 2014
		– Chronic inflammation which favors the progression to AIDS		
miR-tar-3p miR-tar-5p	Viral: HIV-1-derived TAR miRNAs	– Regulation of host gene expression	Fas signaling pathway	Bartz and Emerman, 1999; Ouellet et al., 2013
		– Upregulation of pro-apoptotic proteins		
		– Activation of Fas signaling pathway → complete apoptosis		
		– In early stages of the infection → delay of the viral induced apoptosis		
miR-132	Host	– Downregulated in HBV infection → enhanced carcinogenic feature of HBV	Akt-signaling pathway	Wei et al., 2013; Liu et al., 2015; Chen et al., 2019
		– Normal levels → inhibition of HCC cell proliferation		
miR-372 miR-373	Host	– Upregulated in HBV infection	NFIB-dependent pathway	Guo et al., 2011; Langroudi et al., 2015; Wei et al., 2015; Ghasemi et al., 2018; Ullmann et al., 2018
		– Levels correlated with the number of HBV DNA copies		
		– Proviral functions → increased viral expression and replication during HBV infection		

## Identifying Techniques for MicroRNAs

Developing an efficient, inexpensive method for the detection of miRNAs involved in a way or another in viral infections would be beneficial not only for the diagnostic process of the more serious infectious diseases, but also for discovering new miRNAs previously unknown to be involved in this pathology. However, there are many challenges to finding such a detection method, partially because miRNAs represent a fairly new field of research and as a result many of their characteristics, such as detection levels, concentration in the biological samples, up-/downregulation in healthy and pathological samples yet remain unknown (Gillespie et al., 2018).

By far, the preferred method for the identification of miRNAs is quantitative reverse transcriptase Polymerase Chain Reaction - PCR (RT-qPCR) (Chen et al., 2018; Gillespie et al., 2018; Yuan et al., 2018). The most frequently used PCR quantitation technique is stem-loop reverse transcription (RT)-based TaqMan MicroRNA assay, which provides high sensitivity and specificity (Chen C. et al., 2011). Schematically, this technique requires two steps, the first one being stem-loop reverse transcription, in which the primers bind the 3′ end of miRNA molecules that will further on be reverse transcribed, and a second step for the quantification of microRNAs using real-time PCR (Chen et al., 2005). Other PCR methods available are poly (A) tailing-based and direct RT-based SYBR miRNA assays (Chen Y. et al., 2011). However, the RT-qPCR method is not lacking in disadvantages either, since it presents a high risk of contamination during the amplification steps, as well as of sensing errors for the samples (Gillespie et al., 2018).

Another popular quantitative method for the detection of miRNAs is Northern blot hybridization, which requires the total RNA to be separated on polyacrylamide gel with denaturation proprieties, after which it is transferred to a nylon membrane, UV-cross-linked and finally hybridized with the help of a probe labeled with a radioactive substance (Kruszka et al., 2014; Barciszewska-Pacak et al., 2015; Pacak et al., 2016; Smoczynska et al., 2019). Still, it is a laborious, time-consuming technique which requires high amounts of RNA and can sometimes miss the identification of rare miRNAs (Smoczynska et al., 2019). Recently, new protocols have been developed in order to enhance the technique, leading to the use of lower levels of RNA and to a shortening of the time needed to execute the procedure (Varallyay et al., 2007; Pall and Hamilton, 2008; Wang X. et al., 2010).

*In situ* hybridization (ISH) and next-generation sequencing (NGS) could also be used for the identification of miRNAs. ISH allows the visualization of RNA in fixed tissue samples and the comparison of miRNA expression levels in different cell types using fluorescent, dioxygenin or radioactive probes to bind the targeted RNA (Javelle and Timmermans, 2012). However, this is also a time-consuming, laborious technique, prone to error at each step of the process (Javelle and Timmermans, 2012). NGS is actually a second-generation method of sequencing, following the well-known Sanger technique (Hu et al., 2017). There are various NGS platforms available that also provide kits for miRNAs quantification. The general principle on which they all function is based on the amplification and sequencing of DNA fragments, with additional steps for miRNA sequencing, starting with miRNA extraction after which the miRNAs are reverse transcribed into cDNA (Bar et al., 2008; Creighton et al., 2009; Hu et al., 2017). NGS is able to identify single miRNA with the high resolution of one nucleotide, yet the high cost of this method, compared to the others, limits its accessibility (Smoczynska et al., 2019). However, this technique has numerous other crucial attributes that should be taken into consideration when choosing a quantification method, such as its high throughout, meaning that the samples one researcher sends would be sequenced in the same time with many other samples (Hu et al., 2017). In addition, NGS offers the possibility of discovering new miRNAs, an advantage that is not provided by a hybridization technique, as well as a high accuracy (Hu et al., 2017).

## MicroRna Signaling in Viral Infection

Viruses represent microorganisms that are entirely dependent on a host cell in order to survive, proliferate and perform all the other functions necessary for their life cycle. To achieve this goal, they have evolved a number of mechanisms aimed to elude the immune system of the host.

The first step in every infection, regardless of the type of virus involved, is the entrance of the microorganism into a susceptible host cell, through the binding between the viral proteins found in the virion and the surface molecules of the host cell, which can also influence the tropism of the infection (Marsh and Helenius, 2006; Greber and Puntener, 2009). Upon attaching to the surface of the cell, the virus needs to pass through the membrane into the cytoplasm. This can be achieved through numerous mechanisms, such as the fusion between the host and virus membranes (Sodeik et al., 1997; Maurer et al., 2008), clathrin-dependent pathways or endocytosis (Marsh and Helenius, 2006; Greber and Puntener, 2009). The following steps are now influenced by the type of virus attempting to infect the organism. For example, in the case of DNA viruses, the genome found in the nucleocapsid has to pass through the cytoplasm and to reach the nucleus, where the transcription of the viral genes takes place. However, the maximum size of the free molecules allowed in the cytoplasm is restricted to approximately 500 kDa (Sodeik, 2000, 2002), therefore oversized viruses make use of the motor proteins and cytoskeleton to achieve this desiderate. The replication of the majority of RNA viruses, on the other hand, takes place solely in the cytoplasm (Greber and Way, 2006), with the exception of retroviruses, which, in the nucleus of the host cell, create a DNA provirus using reverse transcription that is ultimately incorporated into the genome of the host, thus leading to a resistant and prolonged infection (Cullen, 2001).

MicroRNAs play a very important role in the modulation systems of the host, therefore inevitably interacting with a variety of viruses (Umbach and Cullen, 2009). There are a number of potential interactions described in literature between the two entities. First of all, the host miRNA could regulate different phases of the viral life cycle, such as translation, by attaching itself to the viral RNA or mRNA. Also, the virus could exert an effect upon the host miRNA, thus leading to an altered expression of the latter’s targets. Moreover, the microorganism could produce its own miRNAs, which would further regulate the viral or host RNA targets (Roberts et al., 2011).

However, indirect roles played by the miRNAs in viral infections have also been described. One such example is the involvement of miRNAs in the modulation of various signaling pathways (Bruscella et al., 2017).

### Signaling Pathways Involved in Viral Infections and the Mechanisms Through Which miRNAs Influence Them

#### Wnt Signaling Pathway

Wnt represents in fact a group of pathways, through which the signal is carried from the extracellular environment into the cytosol and that is evolutionarily preserved in vertebrates. Its name has its origins in a combination between the name of the segment polarity gene of Drosophila (“Wingless”) and the name of its analog found in vertebrates (“Integrated”) (Wodarz and Nusse, 1998). When the Wnt signal reaches the intracellular medium, it triggers several cascades, which are divided as it follows: the canonical pathway or β-catenin dependent and the non-canonical one or β-catenin-independent. The last one can also be subdivided into the Wnt/Ca2 + cascade and the Planar Cell Polarity cascade (Habas and Dawid, 2005). Wnt signaling pathway plays a critical role in cellular growth, polarity, motility and development (Komiya and Habas, 2008).

**FIGURE 1:**
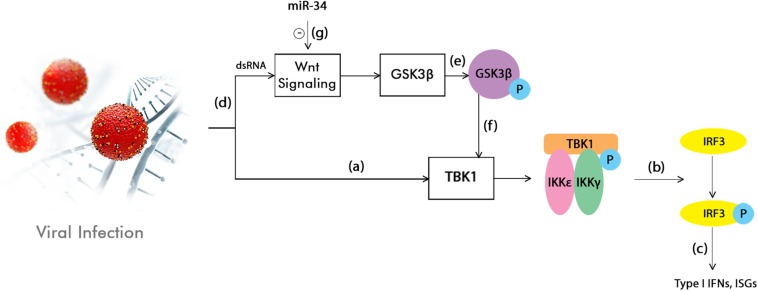
Interferon (IFN) signaling enhancement by the inhibition of the Wnt pathway through miR-34. TANK-binding kinase 1 (TBK1) phosphorylation is firstly induced by the activation of the innate immunity in response to a viral infection **(a)**. This is followed by the phosphorylation or homodimerization of the interferon regulatory factor 3 (IRF3) **(b)**, which is subsequently translocated into the nucleus. Here, it stimulates the production of type I IFNs and interferon stimulated genes (ISGs) **(c)**. At the same time, the viral infection or dsRNA treatments also activate the Wnt signaling pathway **(d)**, which in turn leads to the inhibition of the Glycogen synthase kinase 3 beta (GS3Kβ) phosphorylation **(e)**. This event suppresses the IFN pathway by interacting with TBK1 **(f)**. This model shows that the Wnt signaling pathway has the ability to modulate the innate inflammatory response. By acting as an inhibitor of the Wnt pathway, miR-34 could enhance type I IFN signaling, therefore leading to a cellular antiviral status (**g**; Smith et al., 2017).

One of the miRNA members that has been shown to have an effect on this pathway is the miR-34 family (Kim et al., 2011; Cha et al., 2012; Smith et al., 2017). Previous studies have demonstrated the effect of this miRNA on various flaviviruses subtypes (but not limited to them) and the highly potent inhibition role it plays (Smith et al., 2017). At the same time, it was pointed out that a possible relationship could exists between the Wnt pathway and the innate cellular immune mechanisms (Yang et al., 2010; Zhu et al., 2011; Hack et al., 2012; Baril et al., 2013; Hillesheim et al., 2014; Khan et al., 2015). Because of this discovery and also because of previous observations that miR-34 represses Wnt signaling, theories have emerged stating that type I Interferon (IFN) signaling in viral infections could be enhanced as a result (Smith et al., 2017; Figure 1).

#### Type I Interferon Signaling Pathway

Type I IFN is a very well-known molecule, which has strong antiviral effects. This is achieved by regulating numerous IFN stimulated genes (ISGs), which in turn encode proteins responsible for creating an antiviral state inside the cell (Sen, 2001). The ISG transcription is induced through the activation of Jak/STAT pathway (Darnell et al., 1994). All type I IFNs attach to the same receptor found on the surface of the cell (Interferon alpha/beta receptor - IFNAR), resulting in the activation of the tyrosine kinases Janus kinase 1 (Jak1) and Tyrosine kinase 2 (Tyk2) associated with the receptor. Following the phosphorylation of the kinases, Signal transducers and activators of transcription 1 and 2 (STAT1 and STAT2) are being activated, and then transported to the nucleus, where they attach to various DNA subunits, thus acting as ISGs promoters. The most important role of ISGs is antiviral, but they can also play a part in apoptosis, inflammation, lipid metabolism and protein degradation (de Veer et al., 2001).

Numerous miRNAs have been shown to interfere in this pathway, more precisely by targeting the receptor IFNAR1. For example, they can indirectly regulate the signal delivery by interacting with Suppressor of cytokine signaling 1 (SOCS1), which decreases the activity of JAK-STAT cascade through binding to the TYK2 part of the receptor complex (Piganis et al., 2011). Some of the miRNAs that have been demonstrated to act in this manner are miR-155, miR-19a and miR-122, leading to an increase in type I IFN signaling and consequentially to an enhancement of the innate and adaptive immune reactions (Pichiorri et al., 2008; Jiang et al., 2010; Wang P. et al., 2010; Yao et al., 2012; Li et al., 2013). Also, in the case of miR-155, it has been shown that this type of interaction results in an increased activation of the anti-viral genes, therefore inhibiting the Hepatitis B virus (HBV) replication (Su et al., 2011).

#### NF-κB Signaling Pathway

Nuclear factor κB (NF-κB) signaling pathway is responsible for regulating a number of genes that are essential for the innate and adaptive immunity. Its activation depends on the recognition of pathogen associated molecular patterns (PAMPs) by the pattern recognition receptors (PRRs) encoded by the germ line (Janeway, 1989). NF-κB consists of reticuloendotheliosis protein dimers (Rel) which attach to a DNA sequence called the κB site. There are two types of Rels, divided according to the state that they can be found: mature or immature. The immature ones include NF-κB1 (p105) and NF-κB2 (p100), which turn into p50 and p52, and the mature ones are represented by RelA (p65), RelB and c-Rel. The most abundant form in the majority of dormant cells is the p50-RelA dimer (Ryseck et al., 1995).

The NF-κB signaling pathway can be activated in two different ways (Karin et al., 2002; Karin, 2006). The canonical pathway includes the dimers that contain RelA, p50 or c-Rel, all of which are held in the cytoplasm by κB proteins inhibitors, such as IκBα, IκBβ, IκBγ, IκBε, IκBζ, IκBNS, and Bcl-3. This first pathway is triggered by members of the proinflammatory cytokines, such as Tumor necrosis factor alpha (TNF-α) and, through the toll-like receptor (TLR), targets the β-subunit of the IκB kinase (IKKβ) complex. The non-canonical pathway is activated by TNF cytokines (Lymphotoxin beta - LTβ), whose target is the α-subunit of IKK (IKKα), using the TNF receptor (Ma et al., 2011).

According to previous studies, the expression of the miRNAs implicated in the modulation of NF-κB can be altered by viral infections and also, through targeting the NF-κB pathway, the viral miRNAs could be responsible for the variations of the immune response (Ma et al., 2011). One of the ways miRNAs use to regulate the NF-κB pathway is by controlling the expression of PRRs. For example, it has been shown that Kaposi sarcoma associated herpesvirus (KSHV) and Human immunodeficiency virus (HIV) infections lead to a decrease in the expression of miR-233 and Let-7 family, therefore upregulating the expression of TLR3 and TLR4 and, as a result, increasing the levels of tissue damage and inflammation (Androulidaki et al., 2009). Also, evidence suggest that the expression of miR-146 and miR-21 is increased in HIV and Hepatitis C virus (HCV) infections, leading to a decrease in the expression of Tumor necrosis factor receptor associated factor 6 (TRAF6) and Interleukin 1 Receptor Associated Kinase 1 (IRAK1), thus reducing the NF-κB activity (Bhaumik et al., 2008; Houzet et al., 2008).

#### PI3K/Akt Signaling Pathway

Phosphoinositide 3-kinase/Protein kinase B (PI3K/Akt) Signaling Pathway represents an important option for viruses to influence a variety of cellular functions. One of the most significant ways in which microorganisms can alter the normal life cycle of the cell is slowing down apoptosis, therefore increasing the time for the virus to replicate. As a result, viruses could also facilitate the induction of carcinogenesis. Recently, other roles played by this pathway in the interaction between the virus and the host have been proposed, such as regulation of the cell metabolism, morphology or immune response (Ji and Liu, 2008). Amongst the viruses that have been reported to activate the PI3K/Akt signaling pathway are hepatitis B virus (HBV), human cytomegalovirus (HCMV), human immunodeficiency virus (HIV), human papilloma virus type 16 (HPV16) and hepatitis C virus (HCV) (Deregibus et al., 2002; Kim et al., 2006; Shen et al., 2006; Guo et al., 2007; Alisi et al., 2008; Chugh et al., 2008).

**FIGURE 2:**
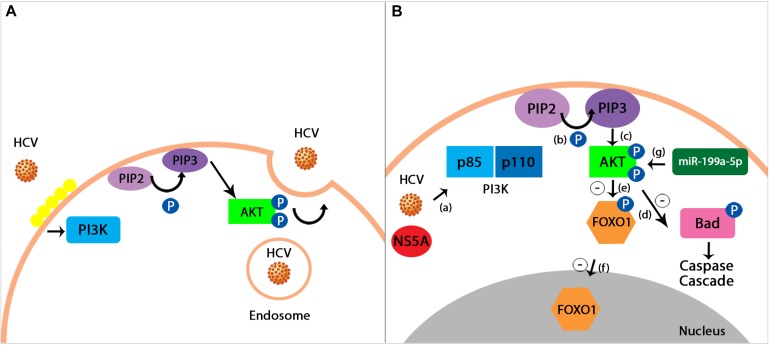
**(A)** HCV entrance into the host cell using PI3K/Akt pathway. Hepatitis C virus uses a pH-dependent way in order to enter the intracellular environment of the host. The virus causes the lipids on the cellular surface to cluster (depicted in the figure through a yellow lining of the membrane), thus triggering the process of endocytosis mediated by the PI3K/Akt signaling pathway (Diehl and Schaal, 2013). **(B)** miR-199a-5p proposed effect on PI3K/Akt pathway during HCV infection. HCV infection activates the PI3K member of the signaling pathway with the help of a viral protein known as Non-structural protein 5A (NS5A) **(a)**. PI3K is a heterodimer formed by the two subunits, p85 and p110. The first one to be phosphorylated is the p85 subunit, followed by p110. This process represents the catalyst of the conversion between phosphatidylinositol 4,5 -bisphosphate (PIP2) and phosphatidylinositol 3,4,5 -trisphosphate (PIP3) **(b)**. PIP3 then activates Protein kinase B (Akt) through phosphorylation **(c)**. Signals produced by the virus in order to survive determine an inhibitory phosphorylation, mediated by the Akt, of the intracellular agents causing apoptosis, such as BCL2 associated agonist of cell death (BAD), which in turn leads to the suppression of the Caspase Cascade **(d)**. Furthermore, the phosphorylation of Forkhead box protein O1 (FOXO1), an important transcription factor **(e)**, stops it from translocating into the nucleus, therefore leading to an inhibition upon the pro-apoptotic genes expression **(f)**. Prior studies have demonstrated the fact that the overexpression of miR-199a-5p plays an important role in enhancing the pro-survival PI3K/Akt pathway. Their results have shown that by decreasing the miR-199a-5p levels, the phosphorylated Akt (p-Akt) levels are also significantly diminished, therefore leading to the proposed mechanism through which miR-199a-5p exerts its actions by promoting Akt phosphorylation (**g**; Diehl and Schaal, 2013; Wang et al., 2015; Adimonye et al., 2018).

MicroRNAs are influenced by the virus-host interaction, in the sense of promoting the survival of the virus. For example, studies have demonstrated that HCV upregulates hsa-miR-483-3p and hsa-miR-320c, which in turn target the PI3K/Akt pathway and enhance the cell survival (Shwetha et al., 2013). Also, the downregulation of miR-199a-5p is responsible for blocking PI3K/Akt signaling in HCV infection, thus inhibiting the replication of the virus (Wang et al., 2015). Even though the exact mechanism is not yet fully understood, prior studies have shown an important reduction in the phosphorylated Akt (p-Akt) levels, leading to the conclusion that PI3K/Akt pro-survival pathway is significantly blocked by knocking down miR-199a-5p (Wang et al., 2015; Figure 2).

#### MAPK Signaling Pathway

MAPK or mitogen activated protein kinase signaling pathway is responsible for converting a variety of extracellular signals into intracellular actions, such as immune response, differentiation, proliferation and apoptosis (Pearson et al., 2001; Dong et al., 2002). In mammals, it can be divided into 3 main pathways: p38 MAPK, SAPK/HNK and MAPK/ERK. The MAPKK is responsible for regulating the activities of these 4 enzymes. For example, MKK3/6 and MKK4/7 are involved in the activation of p38 and JNK (responsible for the expression of cytokines and apoptosis) (Ludwig et al., 2001), while MEK5 activates the enzyme ERK5 (Ludwig et al., 2006). This signaling pathway has been shown to play an important role in viral infections, such as HCV (which increases the activity of p38 MAPK pathway), HIV (which upregulates the ERK activation) (Gaur et al., 2011) or cytomegalovirus (which, by increasing the activity of MAP2K3, maintains p38 active for viral replication) (Johnson et al., 2000).

**FIGURE 3:**
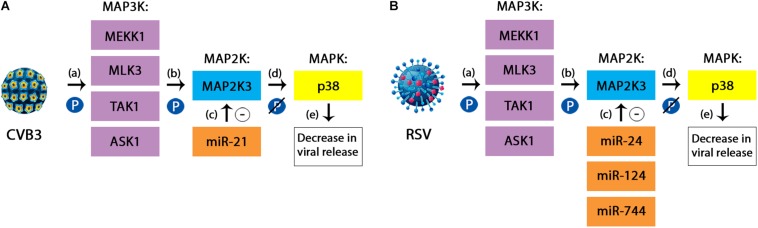
**(A)** The protective mechanism of miR-21 against Coxsackievirus B3 (CVB3). CVB3 is responsible for activating the p38 component of the MAPK signaling pathway during the course of infection. The simplified mechanism relies on three subsequent phosphorylations, targeting the MAP3K members **(a)**, the MAP2K (which in the case of CVB3 is represented by MAP2K3 or MKK3) **(b)** and finally the MAPK, represented here by the p38 kinase. This cascade leads to an increased viral replication in the infected cells. miR-21 has been shown to inhibit the expression of MAP2K3 **(c)**, therefore leading to a reduced phosphorylation of p38 **(d)** and consequently, a decrease in the viral release **(e)**. **(B)** The protective mechanism of miR-24, miR-124, and miR-744 against Respiratory Syncytial Virus. RSV infection follows the same steps in order to activate the p38 MAPK pathway. In this case, the miRNAs involved are miR-24, miR-124, and miR-744. However, besides the decrease in the activation of p38 component due to a lack of phosphorylation, there has also been demonstrated a reduction in the overall levels of the same member (McCaskill et al., 2017; He et al., 2019).

MiRNAs have also been found to participate in this cascade during viral infections. For instance, miR-21 could act as a protective factor during Coxsackievirus B3 (CVB3) infection, by targeting the MAP2K3/p38 MAPK pathway. In this process, miR-21 has been shown to inhibit the expression of MAP2K3, leading to a reduced phosphorylation of the p38 MAPK, thus resulting in a significant decrease in the release of viral progeny (Figure 3; He et al., 2019). Also acting upon the same pathway are miR-744, miR-24 and miR-124, which are considered potential antiviral agents for RSV virus and influenza. In this case, the mechanism seems to be similar to the one described in the case of miR-21 and CVB3, with decreased levels of phosphorylated p38 showing a reduced activation of this component. Furthermore, a significant reduction in the expression of p38 MAPK has also been demonstrated through decreased levels of proteins (McCaskill et al., 2017).

#### Notch Signaling Pathway

The Notch pathway is responsible for the cellular differentiation, proliferation and apoptosis. Notch actually represents a receptor found on the surface of the cell that intercepts nearby signals through its interactions with the transmembrane ligands of neighboring cells, such as Jagged and Delta-like. The Notch intracellular domain (NICD) is then cleaved and released in the intracellular environment, traveling through the Golgi apparatus to the nucleus, where it modulates the transcription of complexes that contain the protein CSL, with the ability to bind DNA (Kopan, 2012).

In viral infections, the ability of this pathway to regulate the differentiation and proliferation of the cell makes it an appealing target for viruses that depend on cellular differentiation, such as HCV, HPV, KSHV, EBV and adenovirus. While HPV enhances the already activated Notch pathway, KSHV and EBV can imitate this type of signaling. However, all of these viruses also have the potential to interfere in the control of the cellular cycle, thus leading to a dysregulated growth (Hayward, 2004). One of the miRNAs that was shown to have an effect on the Notch pathway is miR-449a. *In vitro* studies have demonstrated that in HCV infected patients, miR-449a targets Notch1, which in turn regulates the expression of Chitinase-3-like protein 1 (YKL40). The latter is thought to play an important role in the development of hepatic fibrosis and the control of inflammatory responses (Sarma et al., 2012).

### MicroRNA Signaling in HPV Infection

Human papillomavirus (HPV) is a small virus containing 8kb of double stranded DNA in its genome (Bouvard et al., 2009). Over 150 different HPVs are known so far, out of which 12 are indisputably linked to cancer by the World Health Organization. Probably the most studied are Human papillomavirus 16 (HPV16) and Human papillomavirus 18 (HPV18), mainly because of their frequent association with cancer. Both of them contain two types of genes called “early” (E1, E2, E4, E5, E6 and E7) and “late” (L1 and L2) genes. Even though E1, E2, L1 and L2 are always encountered in all HPVs, the other genes may vary (Stubenrauch et al., 2007; Nobre et al., 2009). The major oncogenes are considered to be E5, E6 and E7, which are also known for altering a variety of signaling pathways, such as Wnt, Notch, PI3K/Akt, MAPK, IFN and NF-κB and others (Gupta et al., 2018; Xu et al., 2018; Wang and Chen, 2019). However, studies have demonstrated that the HPV infection could alter the interaction between miRNAs and some of these pathways.

#### MicroRNA Modulation of PI3K/Akt Signaling in HPV Infection

PI3K/Akt pathway plays an essential role in numerous human cancers associated with HPV. Both E6 and E7 oncogenes activate PI3K/Akt signaling pathway by influencing a number of events, therefore leading to carcinogenesis. In HPV positive laryngeal papilloma, the activity of PI3K/Akt is significantly upregulated, causing the stimulation of the Epidermal Growth Factor Receptor (EGFR) and consequently, the activation of MAPK/ERK cascade (Rodon et al., 2013). Furthermore, the E7 oncogene and its ability to increase the activity of this pathway, has been linked to the inactivation of Retinoblastoma (Rb) protein, leading to high-grade cervical neoplasia (Menges et al., 2006).

One of the miRNAs that affects this pathway due to the HPV infection is miR-125b. Some studies have shown that E6 oncogene downregulates the levels of miR-125b (Ribeiro and Sousa, 2014), which is responsible for the suppression of the PI3K/Akt signaling pathway, therefore limiting the growth of cancerous cells and promoting apoptosis (Cui et al., 2012). However, a more recent study discovered increased levels of miR-125b induced by the overexpression of octamer-binding transcription factor 4 (OCT4) gene, which, in turn, reduced the expression of Bcl-2 homologous antagonist/killer (BAK1) protein, therefore decreasing the apoptosis of cervical cancer cells (Wang et al., 2013). The different findings were explained by the fact that miR-125b has a variety of targets, its roles being influenced by the various levels of expression of their genes (Banzhaf-Strathmann and Edbauer, 2014).

#### MicroRNA Modulation of MAPK/ERK Signaling in HPV Infection

In HPV infection, the MAPK/ERK signaling pathway is activated by the E5 oncogene (Fanger, 1999). Mitogen activated protein kinase (MAPK) signaling pathway is essential for the regulation of cancerous cell invasion and metastasis, primarily because of the role it plays in cellular differentiation, proliferation and apoptosis. Some of the miRNAs that could influence this pathway in the HPV infected organisms are miR-23b, miR329-3p and miR200c. MiR-23b acts as an inhibitor of metastasis in cervical cancer (Li et al., 2018), while miR-329-3p (Li et al., 2017) and miR-200c (Mei et al., 2018) are known for suppressing the migration of the cells and further invasion by targeting MAPK1 and MAPK4.

miR-23b exerts its tumor suppressor function by downregulating the MAPK1 expression, through direct binding to the 3′UTR end of MAPK1. Furthermore, MAPK1 is responsible for mediating the expression of the matrix metallopeptidase 9 (MMP-9), a molecule that has been associated with tumor migration (Nelson et al., 2000). miR-329-3p also binds directly to MAPK1 through a target sequence encountered in its 3′UTR end, leading to a subsequent suppression of the invasion, migration and proliferation of cancerous cells (Li et al., 2017). MAPK4 could involve p38, c-Jun N-terminal kinase (JNK) and extracellular signal-regulated kinase (ERK) signaling pathways in order to accomplish its functions in the malignancy (Bouzakri and Zierath, 2007). It is responsible for cellular proliferation, invasion and metastasis, angiogenesis and apoptosis inhibition. miR-200c binds to MAPK4 through the same direct mechanism, via the 3′UTR region (Mei et al., 2018).

#### MicroRNA Modulation of IFN Signaling in HPV Infection

Signal transducer and activator of transcription (STAT) family is responsible for the survival and progression of HPV associated cervical cancer, in particular STAT3 member, whose aberrant expression has been linked to transforming attributes (Aggarwal et al., 2006). STAT3 carries signals to the target genes via the JAK/STAT signaling pathway. A potential binding site for STAT3 could exist on the Long Control Region (LCR) of HPV16, at the 5′ end, which could regulate the expression of the viral oncogenes (Arany et al., 2002).

Previous studies linked miR-21 and Let-7a with this signaling pathway. Inhibition of STAT3 signaling using pharmacological substances like curcumin resulted in a cellular decrease of miR-21 (Shishodia et al., 2014). Furthermore, the downregulation of miR-21 levels with the help of a miR-21 inhibitor lead to a negative regulation of STAT3 and decreased amounts of active pSTAT3. Apart from this miRNA’s action upon STAT3, Let-7a is another member that has been shown to have an effect on this pathway. An increased level of Let-7a, using a chemically produced analog, decreased the STAT3 supplies. In conclusion, the silencing of the HPV E6 oncogene has been linked to an increase in Let-7a and a decrease in miR-21, therefore downregulating the STAT3 signaling pathway (Shishodia et al., 2014). The inhibition of miR-21 results in elevated PTEN levels, which is a well-known tumor suppressor gene. miR-21 targets this gene through a sequence found on the 3′ UTR. PTEN is responsible for the downregulation of STAT3 activity, while its expression is only partially influenced. The mechanism through which Let-7a downregulates STAT3 involves a direct binding site encountered on the 3′UTR of STAT3 for this particular miRNA (Wang Y. et al., 2010). Let-7a has been demonstrated to suppress the growth of cancerous cells, both *in vivo* and *in vitro* (Trang et al., 2010).

#### MicroRNA Modulation of NF-κB Signaling in HPV Infection

Nuclear Factor κB signaling pathway plays an important role in the progression of HPV induced cervical cancer progression, through its increased DNA binding potential (Prusty and Das, 2005; Branca et al., 2006). In the case of oral cancers caused by HPV16, the excessive expression of E6 and E7 oncogenes and the interaction with the NF-κB pathway lead to a better tumor differentiation, as well as an improved prognosis (Gupta et al., 2018). There were two miRNAs found to interact with this signaling pathway during HPV infection, and more specifically, in cervical cancers: miR-143-3p and miR-218-5p (Xu et al., 2018). Both of them have the mRNA LYN as a target, which was found to be highly expressed in HPV induced cervical cancers, thus increasing the activity of NF-κB pathway. MiR-218-5p acts as a negative regulator of this pathway, by decreasing the expression of LYN (Xu et al., 2018).

### MicroRNA Signaling in Herpes Virus Infection

Herpesviridae is a vast family of enveloped dsDNA viruses that are infectious to both humans and animals (Grey, 2015). Although to date over 130 viruses have been recognized (Brown and Newcomb, 2011), the ones that infect humans are comprised of three subgroups, namely α-herpesviruses, which include herpes simplex virus (HSV) types 1 and 2 and the varicella-zoster virus (VZV), β-herpesviruses which encompass the cytomegalovirus (CMV) and human herpesviruses 6 and 7 (HHV), as well as the γ-herpesviruses, involving the Epstein-Barr virus and Human Herpesvirus-8 (HHV8) (Wagner and Bloom, 1997; Carter, 2007).

#### MicroRNA Signaling in Herpes Simplex Virus Infection

HSV-1 and HSV-2 are most common herpes viruses, infecting individuals mainly through oral and/or genital contact, at the hand of shed viral particles. The virus drifts from the mucosa until it reaches the neighboring autonomic neurons, where it sets up a latent infection (Du et al., 2015). It has long been shown that during latent infection, viral gene expression is limited to the latency-associated transcript (LAT) (Wagner et al., 1988). While this non-coding viral ARN is not fundamental in the latency of infection (Randall et al., 2000), it does play a role in latency-associated processes such as establishment, maintenance and reactivation, arguably by means of LAT-encoded miRNAs (Perng et al., 1994; Umbach et al., 2009; Nicoll et al., 2012).

HSV-1 and -2 encode 18 precursor miRNAs that are responsible for ultimately producing 27 and 24 mature miRNAs, respectively (Kozomara et al., 2018). Despite efforts to attribute exact functions to these miRNAs (Tang et al., 2009; Duan et al., 2012), they still remain largely unexplained, with most studies suggesting their involvement in latency regulation (Piedade and Azevedo-Pereira, 2016). To this extent, HSV miR-H3 and miR-H4 are two miRNAs that have been shown to decrease the expression of the infected cell polypeptide 34.5 (ICP34.5), a protein that promotes viral replication and reactivation in neurons (Tang et al., 2008; Tang et al., 2009). Moreover, when targeting the p16 protein, miR-H4b inhibits the PI3K/Akt and mTOR signaling pathways, therefore modifying the cellular environment in order to better adapt it for viral replication and latency (Gottwein and Cullen, 2008; Zhao et al., 2015). Along the same lines, miR-H6 has also been shown to promote the maintenance of a latent state of HSV-1 by targeting the Infected-cell polypeptide 4 (ICP4), which normally pushes the virus toward entering the lytic cycle (Tang et al., 2009; Duan et al., 2012). Infected-cell polypeptide 0 (ICP0) is another protein that stimulates the reactivation of HSV-1, as well as viral replication (Everett, 2000), and it has been shown that its activity is hindered by miR-H2 (Umbach et al., 2008). Recently identified miR-H28 and miR-H29, expressed late during reactivation, also tend to reduce excessive viral replication (Han et al., 2016). It was later demonstrated by Huang et al. that only miR-H28 induces IFNγ in human cell lines in order to limit the viral spread to uninfected neighboring cells. They showed that both miR-H28 overexpression and IFNγ induction happen at the same time, at the end of the reproductive cycle. Their study found increased levels of IFNγ at 7 h and, most notably, at 18 h after transfection with miR-H28 (Huang et al., 2019). Han et al. had previously suggested that IFN could be activated at the hand of the stimulator of interferon genes (STING) member of the signaling pathways, which is exported through exosomes from infected cells to healthy ones (Han et al., 2016). It is hypothesized that HSV tends to decrease its replication in order to avoid its spread to the central nervous system, which would in all likelihood kill the host (Han et al., 2016). On the other hand, several viral miRNAs have been shown to accumulate during viral replication and reactivation, such as miR-H8-5p, -H15, -H17, -H18, -H26, and -H27, leading to speculation regarding their involvement in promoting these processes (Du et al., 2015).

#### MicroRNA Signaling in Cytomegalovirus Infection

Cytomegalovirus is also characterized by the establishing of life-long latency following infection resolution, and poses great risks for immunocompromised patients (Varani and Landini, 2011). To date, the miRBase database reports the identification of 26 mature human CMV miRNAs originating from 15 precursor miRNAs (miRBase, 2018). Their main functions are thought to be immune system evasion, cell cycle regulation and vesicular transport (Hook et al., 2014).

Hook et al. have shown that HCMV miRNAs UL112-1, US5-1, and US5-2 target different components of the secretory pathway, among which are the Ras-related protein Rab-11A (RAB11A), the vesicle-associated membrane protein 3 (VAMP3) and the synaptosome-associated protein of 23 kDa (SNAP23) (Hook et al., 2014). Under normal conditions, these endocytic proteins facilitate the release of Interleukin-6 (IL-6) and Tumor necrosis factor alpha (TNF-α), whose suppression permits a bypass of the innate immune response (Gealy et al., 2005; Jarvis et al., 2006). Moreover, miR-UL112 has also been shown to decrease the natural killer (NK) cell recognizing of the virus-infected cells by targeting the major histocompatibility complex class-I related chain B (MICB) (Stern-Ginossar et al., 2007). A thought-provoking observation was developed by Nachmani et al., when they found that this viral miRNA acted in synergy with a host miRNA, hsa-miR-376a, thus decreasing NK-mediated virally infected cell killing (Nachmani et al., 2010). A diminishing in the recognition by the NK cells has also been observed to be determined by miR-US25-2-3p, a viral miRNA that also targets the tissue inhibitors of metalloproteinase 3 (TIMP3), leading to an increase in the shedding of MICA/B (Esteso et al., 2014). Furthermore, two fairly newly discovered HCMV miRNAs, miR-US5-1 and miR-UL112-3p, have recently been shown to target members of the IκB kinase (IKK) complex, IKKα and IKKβ, therefore reducing NF-κB signaling during late infection (Hancock et al., 2017) and, consequently, the expression of proinflammatory genes encoding cytokines and chemokines (Liu et al., 2017). The secretion of cytokines and chemokines including IL-6 is further inhibited by miR-UL148D, viral miRNA that has been shown to be predominantly expressed during latency (Lau et al., 2016; Goodwin et al., 2018).

#### MicroRNA Signaling in Epstein Barr Virus Infection

Much the same as other herpesviruses, EBV also causes latent life-long infection by means of episomes maintained within memory B-lymphocytes (Babcock et al., 1998; Thorley-Lawson et al., 2013), its persistence having been associated with lymphoproliferative disorders such as Burkitt’s lymphoma, Hodgkin’s lymphoma, and diffuse large B cell lymphoma (Shannon-Lowe et al., 2017).

EBV miRNAs play a number of roles in its pathogenesis, including apoptosis inhibition - BART miRNAs (Marquitz et al., 2011), immune evasion - BHRF1-3 (Xia et al., 2008), BART2-5p (Nachmani et al., 2009), BART15 (Haneklaus et al., 2012) and, perhaps the most crucial in tumorigenesis, cellular proliferation and transformation (Wong et al., 2012; Lei et al., 2013), mainly by modulating the expression of tumor suppressor genes. On this note, miR-BART3 has been shown to inhibit the activity of deleted in cancer 1 (DICE1) protein, therefore counteracting its tumor suppressive activity (Lei et al., 2013). Moreover, EBV miR-BART7-3p has been found to regulate the PI3K/Akt/GSK-3β pathway by targeting the phosphatase and tensin homolog (PTEN) tumor suppressor gene. This leads to an accumulation of Snail and β-catenin proteins, whose degradation is further inhibited by the suppression of the beta-transducin repeat containing E3 ubiquitin protein ligase gene (BTRC) mediated by miR-BART10, thus favoring epithelial mesenchymal transition (EMT) and metastasis in nasopharyngeal carcinoma (Cai L.M. et al., 2015). A similar mechanism of action involving the decrease of PTEN activity followed by the activation of PI3K-Akt, FAK-p130^Cas^ and Shc-MAPK/ERK1/2 signaling pathways has been attributed to miR-BART1 in nasopharyngeal cancer cells (Cai L. et al., 2015). Moreover, miR-BART16 has been shown to target the CREB-binding protein (CBP), a key factor of the IFN signaling pathway (Hooykaas et al., 2017). By impairing the IFN pathway, EBV not only ensures its own replication, but it also damages the anti-tumor effects of IFN (Budhwani et al., 2018). On the other hand, inhibitory genes of the Wnt signaling pathway can be inhibited by EBV miRNAs such as miR-BART-19-3p (targets WNT Inhibitory Factor 1, WIF1), miR-BART7, 17-5p, and 19-3p (targets the adenomatous polyposis coli gene, APC) and miR-BART14, 18-5p, 19-3p (targets nemo-like kinase, Nlk) (Wong et al., 2012). Similar to the impairing of PI3K/Akt/GSK-3β pathway by miR-BART7-3p, an aberrant regulation of the Wnt pathway causes a shift of cytoplasmic β-catenin into the nucleus, which ultimately leads to excessive cellular proliferation (Wang et al., 2017).

### MicroRNA Signaling in HIV Infection

The human immunodeficiency viruses (HIV) are RNA viruses belonging to the Retroviridae family and consist of two genetically distinct types: HIV-1, the more common and more virulent one, and HIV-2, which is mainly limited to the West African territory and is associated with lower transmission rates and forms of disease (Gilbert et al., 2003). HIV-1 infection gradually leads to a decrease in T-cell CD4^+^ cells, therefore weakening the patient’s immune defense, making them susceptible to many infections. The means by which HIV manages to evade the host’s immune strategies involves the reshaping of the host cells with the aid of viral accessory proteins and RNA (Fruci et al., 2017). However, while host miRNAs can play either positive or negative roles in viral replication and disease evolution (Balasubramaniam et al., 2018), the existence of viral miRNAs has only relatively recently been demonstrated and their role in pathogenesis is still disputed (Omoto et al., 2004).

#### Cellular MicroRNAs Involved in Cell Signaling Pathways During HIV Infection

A crucial step in HIV infection is the maintenance of latency, which is facilitated by transcription, a process which involves various cellular and viral factors, perhaps the most important of which is the viral transcriptional activator Tat (Asamitsu et al., 2018). A recent study pointed out that hsa-miR-21 and -222 upregulation mediated by Tat may offer protection against apoptosis while also leading to anergy in infected CD4^+^ T cells. This is made possible due to the impairment of the PTEN-AKT-FOXO3a pathway in infected cells, which leads to the inhibition of PTEN-mediated apoptosis (Sanchez-Del Cojo et al., 2017). Furthermore, Sardo et al. have shown that, when binding to certain Dicer proteins, Tat downregulates particular cellular miRNAs, specifically miR-539, -135a, -129, -499, -523, -524, 181a, and let-7. This, in turn, interferes with the Wnt/β-catenin pathway, with Tat lysine motifs at positions 41 and 51 playing crucial yet still poorly understood roles in inducing the suppression of the β-catenin activity. The developing of HIV-associated neurocognitive disorders is consequently promoted, the Wnt/β-catenin pathway being just one of the pathways involved in their pathogenesis (Sardo et al., 2016). An important defense mechanism employed by eukaryotic cells is the RNAi pathway, which has been shown to play an important role in HIV infection (Scarborough and Gatignol, 2017). Nef is an accessory protein that plays a key role in both viral replication and downregulation of CD4^+^ T cells (Lundquist et al., 2002), and Omoto et al. were the first to demonstrate that Nef-shRNA decreased the transcription of HIV-1, thus implying that Nef-miRNAs produced by infected cells have a blocking action on Nef activity through the RNAi pathway (Omoto et al., 2004).

An interesting finding was further highlighted by Xue et al. when looking at HIV-associated Kaposi sarcoma: they found that the HIV-1 Nef and KSHV K1 oncoprotein acted in synergy and upregulated miR-718. Using a dual-luciferase reporter assay system, they managed to show that, of the nine tested miRNAs which were positively expressed when transduced with K1 and/or Nef, only miR-718 directly targeted PTEN. By attaching to a specific binding site on PTEN 3′UTR luciferase reporter, miR-718 decreased PTEN’s tumor suppressor activity in a dose-dependent manner, thus activating the AKT/mTOR signaling pathway and therefore promoting cell proliferation and angiogenesis (Xue et al., 2014). On the same note, Yan et al. have found that, in patients infected with both HIV-1 and KSHV, miR-942 and -711 were upregulated, both leading to an activation of the NF-κB signaling pathway, which, in turn, inhibited KSHV lytic replication (Yan et al., 2018). The NF-κB signaling network has previously been shown to be consistently activated during viral infections, promoting an inflammatory state as well as HIV persistence due to its ability to activate the viral transcription (Hiscott et al., 2001). During HIV infection, it has been shown to be influenced by both cellular and viral miRNAs (Gao et al., 2014). miR-146a/b, which is increased during viral infections in general (Taganov et al., 2006), and HIV in particular (Huang et al., 2018), has been shown to downregulate the NF-κB pathway by targeting the TNF receptor-associated factor 6 (TRAF6) (Paik et al., 2011). Furthermore, higher levels of miR-155 have also been found in infected cells. By targeting the tripartite motif containing 32 (TRIM32) it has been found to block the ubiquitination of IκBα, thus inhibiting the NF-κB pathway and, as a result, promoting viral latency reestablishment (Ruelas et al., 2015).

#### Viral MicroRNAs Involved in Cell Signaling Pathways During HIV Infection

On the other hand, viral miRNAs are also important players in persistent inflammation and disease progression. Hiv1-miR-88 and hiv1-miR-99 have been shown to activate the TLR8 signaling pathway resulting in a steady release of macrophage TNFα (Bernard et al., 2014), which ensures a chronic state of inflammation that ultimately favors progression to AIDS (Silvestri and Feinberg, 2003). Furthermore, HIV-1-derived TAR miRNAs, miR-tar-3p and miR-tar-5p, have been proposed to regulate host gene expression. Ouellet et al. elicited the assumption that TAR miRNAs, by acting on mRNA, regulate apoptosis-related genes, thus impacting HIV-induced cell death (Ouellet et al., 2013). To this extent, an upregulation of pro-apoptotic proteins as a result of these viral miRNAs has been observed, which ultimately activate the Fas signaling pathway, leading to complete apoptosis (Bartz and Emerman, 1999). On the other hand, during the early stages of infection, TAR miRNAs could also delay the viral induced apoptosis in order to gain time for viral replication and assembly (Ouellet et al., 2013).

### MicroRNA Signaling in HBV and HCV Infection

Viral hepatitis is an important public health concern that is mainly caused by the hepatitis B (HBV) and C (HCV) viruses. Chronic infection leads to persistent liver inflammation and damage, which may ultimately end up in hepatocellular carcinoma (HCC) due to the development of oncogenic changes (Shih et al., 2016). To date, there is mounting evidence attesting to the importance of aberrant miRNA expression in the pathogenesis of viral hepatitis.

#### MicroRNA Signaling in HBV Infection

Hepatitis B virus is a DNA virus belonging to the Hepadnaviridae family that causes acute hepatitis which, in around 5% of cases, proceeds to chronic disease (Mui et al., 2017). In some cases, patients suffering from HBV may in time develop hepatocellular carcinoma (HCC) due to the chronic inflammatory state that it ensures and the pro-tumorigenic pathways that it activates (Levrero and Zucman-Rossi, 2016).

While the presence and function of HBV-miRNAs has only been speculated on, multiple studies have highlighted the importance of the impaired expression of cellular miRNAs during HBV infection and oncogenesis. MiR-122 is a liver specific miRNA that plays an important role in cholesterol metabolism, tumor suppression and the maintaining of an overall healthy liver (Jopling, 2012). Wang et al. have shown that HBV replication is negatively influenced by miRNA-122, which increases p53 activity through the downregulation of cyclin G1. Therefore, loss of miR-122 expression has been shown to promote viral persistence as well as oncogenesis (Chen Y. et al., 2011; Wang et al., 2012). Furthermore, Xu et al. have demonstrated that miR-122 has a pronounced antitumor effect by regulating the Wnt/β-catenin pathway. Their experiment indicated that miR-122, by binding to 3′-UTR Wnt1 which included the target sequence, managed to inhibit its activity by almost 50%, this suppressive effect being absent when a miR-122 inhibitor was used. This blocking activity subsequently led to hindering of HCC cell proliferation and promotion of cell apoptosis (Xu et al., 2012). Furthermore, it has been suggested that miR-122 can actively prevent the epithelial-mesenchymal transition while blocking HCC cell motility due to its ability to impair the RhoA/Rock pathway, which is deeply involved in cytoskeletal events such as fiber bundles formation (Kalluri and Weinberg, 2009; Wang et al., 2014). To this extent, Wang et al. tested this hypothesis by using dual luciferase reporter plasmids (RhoA and Rac1) containing the binding sites for miR-122. Their experiment showed that miR-122 significantly diminished the number of total and active forms of RhoA and Rac1 (Wang et al., 2014). Moreover, an inhibition of HBV replication is possible through the regulation of IFN signaling pathway: Gao et al. have shown that the IFN circuitry can be hindered by the overexpression of the suppressor of cytokine signaling 3 (SOCS3), which happens as a result of a down-regulated miR-122 (Gao et al., 2015).

miR-132 has been shown to associate tumor suppressive properties in various types of cancer (Liu et al., 2015; Chen et al., 2019). However, during HBV infection, the hepatitis B virus x (HBx) protein downregulates its expression, thus enhancing HBV’s carcinogenic feature. Wei et al. have demonstrated that HCC cell proliferation was markedly inhibited by miR-132, which interfered with the Akt-signaling pathway. After transfection of tumor cells with miR-132, they found that p-Akt, cyclin D1, p-GSK3β and β-catenin were substantially under-expressed, this outcome indicating the involvement of the Akt signaling cascade and miR-132 in HCC tumorigenesis (Wei et al., 2013).

The miR-371-373 cluster can act either as tumor-suppressors (Langroudi et al., 2015; Ullmann et al., 2018) or potential oncogenes (Wei et al., 2015; Ghasemi et al., 2018) in various human malignancies. In HBV infection, the upregulation of miRs-372-373 has been shown to positively correlate with the number of HBV DNA copies. Guo et al. have highlighted that, by targeting an NFIB-dependent pathway, miR-372-373 promote HBV expression and replication, thus favoring viral progression (Guo et al., 2011).

#### MicroRNA Signaling in HCV Infection

Hepatitis C virus (HCV) is an RNA virus belonging to the Flaviviridae family, which, as opposed to HBV, causes chronic viral hepatitis in around 60-80% of infected individuals (Rehermann and Nascimbeni, 2005). Around 5 to 20% of chronic hepatitis C patients proceed to develop cirrhosis or HCC (Chen and Morgan, 2006). The RNA-dependent RNA polymerase (RdRp) of HCV operates in an error-prone manner which results in a highly diverse population of viral quasispecies (Gomez et al., 1999). The ensuing heterogeneity of HCV poses great challenge not only in developing new treatments and effective vaccines, but also in diagnosis (Li and Lo, 2015).

While miRNAs impaired by HBV are mainly involved in DNA damage and repair, transcription and apoptosis, those suppressed by HCV have been shown to have an impact mainly on antigen presentation, immune regulation and cell-division cycle. Moreover, it would seem that in HCV infection there are more down-regulated miRNAs than in HBV infection (Ura et al., 2009), with Jopling et al. suggesting that some cellular miRNAs may be depleted during the defense against RNA viruses (Jopling et al., 2005). For instance, Huang et al. have shown that Wnt1 acts as a target gene, holding two binding sites for miR-152, situated at the 3′-UTR region of Wnt, namely at 262–268 bp and 688–694 bp, respectively. They indicated that the expression of miR-152, which normally has an inhibitory effect over Wnt1, was notably downregulated in HCV infection, attributing it to the overexpression of HCV core protein. The ensuing increased activity of the Wnt/β-catenin pathway promotes excessive cell proliferation (Huang et al., 2014). Wnt/β-catenin signaling has also been shown to be increased by the overly expressed miR-155 that is seen in HCV infection, as suggested by Zhang et al. (Zhang et al., 2012). In Wnt signaling, the glycogen synthase kinase 3 beta (GSK3b) enzyme, along with the adenomatous polyposis coli (APC) and Axin proteins assemble a multimeric structure meant to abolish Wnt signaling by phosphorylating β-catenin at its N-terminal end which leads to its degradation through the ubiquitin proteasome system (Verheyen and Gottardi, 2010). In their experiment, miR-155 significantly decreased APC levels, thus leading to the activation of the Wnt/β-catenin signaling cascade (Zhang et al., 2012).

However, HCV infection also results in the upregulation of certain miRNAs, and such is the case of miR-21, which is upregulated by HCV. MiR-21 acts by targeting elements of the toll-like receptor (TLR) signaling pathway, like interleukin-1 receptor-associated kinases (IRAK) 1 and 4, tumor necrosis factor receptor-associated factor 6 (TRAF6) and myeloid differentiation primary response (MyD88) 88 protein, ultimately leading to IFN type I suppression in order to evade the immune response (Chen et al., 2013). Liver-specific miRNA, miR-122, can also be up-regulated during HCV infection, predicting a poorer response to IFN therapy (Khan et al., 2015). Enhanced miR-122 tends to promote the expression of the suppressor of cytokine signaling 3 (SOCS3) and therefore reduce STAT3 activation, thus reducing the induction of antiviral genes through the interferon-stimulated gene factor 3 (ISGF3) (Zhu et al., 2011). IFN resistance is further aided by miR-373, which targets IFN-regulatory factor 9 (IRF9) and JAK1, therefore impairing the JAK/STAT signaling pathway (Mukherjee et al., 2015).

## Clinical Application of Mirnas in Viral Infection and Therapy

In the past years, using miRNA as a tool in order to modify gene expression became one of the most important and new frontier in modern medicine (Chakraborty et al., 2017). Recently, an increased significance has been granted to the field of infectious diseases. MiRNAs are interesting molecules with reference to antiviral therapy because of their low immunogenicity and their capability of being tests in various animal models in preclinical studies (Girardi et al., 2018). Even if there are many ways to inhibit (Li and Rana, 2014) or to overexpress a given miRNA (Yang, 2015) *in vivo*, the delivery process still remains challenging for miRNA-based therapy in clinical applications (Girardi et al., 2018).

An interesting example of miRNA-based treatment for antiviral therapy is Miravirsen, an LNA-modified anti-miR-122 which can combat hepatitis C virus infection (Janssen et al., 2013). LNA is a 15-nucleotide locked nucleic acid oligonucleotide which suffered phosphonothioate modifications and was named SPC3649 or Miravirsen. It was shown that the systematic delivery of LNA, complementary to the 5′ end of miR-122, results in sequestration of miRNA in non-human primates with no associated toxicity (Elmen et al., 2008). Also, in chimpanzees with chronic HCV infection the silencing of miR-122 by the antisense oligonucleotide Miravirsen was also achieved, and long lasting viral suppression was observed (Lanford et al., 2010). 3 years later, Janssen et al. carried out a second phase of the study in chronic HCV infected patients who received 5-weekly injections of Miravirsen, and they observed that the treatment resulted in a prolonged and dose-dependent reduction in HCV RNA levels (Janssen et al., 2013; van der Ree et al., 2014; Kreth et al., 2018). More recently, the evaluation of miR-122 plasma levels in chronic HCV infected patients during Miravirsen treatment demonstrated a specific, significant and prolonged decrease in miR-122 expression, very close to detection limits in some cases. Despite this fact, a substantial decrease in viral load was not observed (van der Ree et al., 2016). The reduction in the viral load was obtained later, in another experiment conducted by van der Ree et al., when RG-101, an N-acetylgalactosamine conjugated antisense oligonucleotide for miR-122 was elaborated in order to increase miR-122 sequestration. Viral load reduction was observed in all treated patients within 4 weeks (van der Ree et al., 2017).

In another study performed on mice, it was demonstrated that the administration of five chemically modified miRNA mimics via intranasal pathway was able to target the viral RNA, suppressing H1N1 replication and protecting the mice from viral infection. The miRNA mimics corresponded to highly expressed miRNAs in respiratory epithelial cells (Peng et al., 2018).

Furthermore, the neurotropic virus JEV is able to induce miR-301 expression in neuronal infected cells, which affects the antiviral host response. *In vivo*, inhibition of miR-301 by intracranial injection with modified miR-301a phosphorodiamidate morpholine oligomer restores the IFN response and improves the survival of JEV infected mice by enabling IFNβ production and restricting viral propagation (Hazra and Kumawat, 2017). Even if miRNAs are a very promising source for the treatment of neurotropic viral infection, the main difficulty for small RNA-based approaches in lies in the crossing of the blood-brain barrier.

One of the most successful interventions nowadays available are live attenuated vaccines against human viral pathogens. Vaccines not only function well for acute diseases, but also for chronic infections such as HIV, even if these are more challenging for reasons of safety and efficacy (Minor, 2015). MiRNAs have a natural capacity to inhibit viruses through direct targeting of viral RNAs, capacity which can be used to generate new attenuated vaccines in specific tissue manner by incorporating cell-specific miRNA target sequences into their genomes. For example, the insertion of complementary sequences for the neural-specific miR-124 into the poliovirus genome restricts its tissue tropism in mice while also preventing pathogenicity of the attenuated viral strain (Barnes et al., 2008).

Another interesting approach refers to the modification of viral tropism in order to develop safer replication-competent oncolytic viruses (Kaufman et al., 2015). It is known that oncolytic viruses replicate in cancer cells and, in turn, trigger the activation of immune response against the tumor, but they can also induce toxicity in normal tissues. To impair this issue, an alternative way is to integrate target sequences complementary to a specific miRNA into the viral genome. The replication in normal cells is reduced while the oncolytic potential is maintained in tumor cells. The oncolytic picornavirus Coxsackie A21, the cause for lethal myositis in tumor-bearing mice, is such an example. The myotoxicity was reduced and the oncolytic properties were maintained when binding sites for the muscle-specific miR-206 and miR-133 were added (Kelly et al., 2008).

## Conclusion

MiRNAs are evolutionarily conserved non-coding RNAs that play crucial roles in regulating gene expression in both animals and humans. In this review, we have extensively covered various aspects involving miRNAs interactions with various signaling pathways during viral infections. It is important to keep in mind that some viruses encode their own miRNAs, which complicates the regulation mediated by the virus.

The importance of these viral small molecules during infection comes from their ability to modify the cell environment in a non-immunogenic way once they are selected by the virus. The overexpression of miRNA triggered by pathogens is not always correlated with their survival or pathogenesis, and sometimes it can be cell- or tissue-specific. The exact mechanisms of modulation of host cellular miRNA by viruses and specific virulence factors are still unclear. However, miRNAs can play important roles in clinical applications in diagnostic and therapy against viral infections.

MiRNAs are essential mediators of host response to different pathogens. Knowing the roles of miRNAs in host response to various viral infections provides an interesting tool for identifying key genes and pathways that must be activated, enhanced, silenced or repressed in order to impel an effective immune response. The clinical applications of miRNAs are extremely important, as miRNAs targeted inhibition may have substantial therapeutic impact. Inhibition of miRNAs can be achieved through many different methods, but chemically modified antisense oligonucleotides have shown the most prominent effects. Though we are far from completely understanding all the molecular mechanisms behind the complex cross-talks between miRNA pathways and viral infections, the general knowledge is increasing on the different roles played by miRNAs during viral infections.

## Author Contributions

MB, CC, and DT contributed to the conceptualization. DC and OT performed the methodology. OB investigated the data. NS carried out the resources. MB, CC, DT, and OB wrote the original draft of the manuscript. DC contributed to writing, reviewing, and editing and supervised the manuscript. OT visualized the data. DT carried out the project administration. NS and SV performed the funding acquisition.

## Conflict of Interest

The authors declare that the research was conducted in the absence of any commercial or financial relationships that could be construed as a potential conflict of interest.
